# Timing, extent and outcomes of public health measures in the first wave of the COVID-19 pandemic in Israel and a comparative analysis by socioeconomic indices

**DOI:** 10.1186/s13584-022-00549-2

**Published:** 2023-01-30

**Authors:** Amit Ginzburg, Deborah Barasche-Berdah, Orly Manor, Ronit Levine-Schnur, Ora Paltiel, Hagai Levine

**Affiliations:** 1 Braun School of Public Health and Community Medicine, Hadassah Medical Organization, Faculty of Medicine, Hebrew University of Jerusalem, Jerusalem, Israel; 2 Harry Radzyner Law School, Reichman University, Herzliya, Israel

**Keywords:** COVID-19, Public health measures, Social distancing, Policy

## Abstract

**Background:**

Early in the coronavirus disease 2019 (COVID-19) pandemic, governments implemented exceptional public health measures (PHMs) in the face of uncertainty. This study aimed to compare mitigation policies implemented by Israel and their timing in the first wave of the pandemic to those of other countries, and to assess whether country characteristics such as democracy, trust, education, economic strength and healthcare reserve were associated with decision-making.

**Methods:**

PHMs and pre-pandemic characteristics, using internationally accepted indices, of 50 countries were collected from 1/1/2020-30/06/2020; and associations between them were assessed. Time to implementation of these measures was compared among the Organisation for Economic Co-operation (OECD) nations. Log-rank test was used for univariate analysis. Cox regression was performed to assess the independent contribution of pre-pandemic characteristics to time-to-implementation of measures. Correlations between timing of specific measures and COVID-19 mortality at 60 days were assessed.

**Results:**

Israel ranked in the upper third of the OECD in swiftness to implementation of eight of the ten measures compared. In univariate survival analysis, countries with an education level below the OECD median were more likely to implement a lockdown (p-value = 0.043) and to close restaurants and entertainment venues (p-value = 0.007) when compared to countries above the OECD median. In Cox regression models, controlling for geographic location**, **democracy level above the OECD median was associated with a longer time-to-implementation of a lockdown (HR=0.35, 95% CI=0.14-0.88, p-value=0.025). Similarly, a high level of GDP per capita was inversely associated with closing schools; and a high level of education inversely associated with closure of restaurants and entertainment venues. Earlier initiation of all PHMs was associated with lower mortality at 60 days, controlling for geographic location.

**Conclusions:**

Israel's initial response to the pandemic was relatively quick, and may have been facilitated by its geographic isolation. Countries with lower pre-pandemic socio-economic indices were quicker to initiate forced social distancing. Early initiation of PHMs was associated with reduced mortality in the short run. Timing of initiation of measures relative to the country-specific spread of disease is a significant factor contributing to short-term early local pandemic control, perhaps more than the exact measures implemented. It is important to note that this study is limited to the initial pandemic response. Furthermore, it does not take into account the broader long-term effects of certain PHMs, which should be a focus of further research.

**Supplementary Information:**

The online version contains supplementary material available at 10.1186/s13584-022-00549-2.

## Background

Early in the course of the coronavirus disease 2019 (COVID-19) pandemic, in the absence of a vaccine or pharmacological interventions, countries responded with “classic” public health measures (PHMs). These measures included isolation of suspected patients and contacts, regional quarantines, and various means of social distancing [1–3]. Countries initiated these PHMs to different degrees and with varying speed in order to contain or slow the pandemic. Strict social distancing measures implemented in China included a complete lockdown of Wuhan, then the epicenter of the pandemic. Most European nations did not enforce social distancing measures until early to mid-March 2020, at which point the virus was widespread in their communities [4]. While most countries adopted social-distancing measures, they varied in two key aspects: timing and intensity [4, 5].

Countries operated in extreme uncertainty as little was known in the early months about the novel coronavirus’s virulence, transmission mode, infection rate or vulnerable populations. Amid this uncertainty, governments were forced to make extreme public health decisions.

After nearly two years of the COVID-19 pandemic, the importance of PHMs in early pandemic containment is clear, though uncertainty remains as to the effectiveness of specific measures and the long-term effects. This can partially be attributed to the difficulties in conducting between-country comparisons due to the many confounding variables [6]. Despite these difficulties, there is evidence that timing of social distancing measures, including stay-at- home orders and school closures, may be an important factor in their effectiveness [7–9]. A recent meta-analysis found that physical distancing as a package of measures was associated with reduced incidence COVID-19, though specific measures such as lockdowns, border closures and school closures could not be assessed due to the high heterogeneity between the studies [10]. There is sparse literature behind the factors influencing decision-making in a pandemic [8, 11–13]. Our objectives were to: (1) compare the extent and timing of the initial PHMs taken by Israel and other countries during the first wave of the COVID-19 pandemic;(2) assess the relationship between country characteristics and PHMs taken; and (3) study correlations between mitigation strategies and their timing with infection-related mortality.

## Methods

### Study design, study population

This is an ecological study, in which countries were the units of analysis. We included 50 countries in the analysis, of which the 38 members of the Organisation for Economic Co-operation (OECD) formed the basis of our study population due to the reliability of their reported data. We supplemented data on 12 other countries based on geographic location, population size and prominence in the pandemic timeline, and geographic dispersion, to complete a global view. These additional countries are Argentina, Brazil, Egypt, Hong Kong, India, Russia, Rwanda, Singapore, South Africa, Taiwan, Thailand, and Uganda. We excluded China from our comparative analysis because of the uniqueness of its pandemic timeline, being the country in which the pandemic originated.

### Data collection, variables and definitions

For our pre-pandemic country characteristics, we utilized commonly used socio-economic and health system indicators from reputable sources as specified below. After excluding highly inter-correlated indicators using a correlation matrix, we chose a single indicator from each of the following categories: *social/political*- this indicator is based on the Economist Intelligence Unit’s (EIU) democracy index. It is based on five categories: electoral process and pluralism; the functioning of government; political participation; political culture; and civil liberties. The final index gives a score of 0–10 and classifies countries as one of four regime types- “full democracy”, “flawed democracy”, “hybrid regime” or “authoritarian regime” [14]. *Economic*- we consider the strength of a country’s economy based on its gross domestic product (GDP) per capita adjusted for purchasing power parity. This indicator is taken from the World Bank (WB) [15]. *Education*-a WB indicator to compare countries’ levels of education is the percent of adults above the age of 25 with upper secondary education [16]. *Healthcare reserve*- we consider the number of hospital beds per 1,000 residents as an indicator of healthcare capacity. This data was collected from the OECD database [17] and Our World in Data (OWID) [18]. Lastly, we considered the level of *trust* in a country as the percentage of people who feel that “most people can be trusted”*-* based on the World Value Survey [19]. The WB does not provide data on Taiwan, therefore Taiwan’s data were collected from additional sources [20–22]. Each of the five indicators was recorded as a continuous variable in its original form. We further divided the study population to “high” and “low” dichotomous scores on these scales based on values above and below the OECD median, respectively.

Data on the different PHMs implemented were recorded for all 50 countries. Ten PHMs were defined as binary variables and the dates of decision and implementation were collected. The measures included: primary school closures, secondary school closures, selective border restrictions, complete border closure, cancellation of mass events, restriction of social gatherings to between 10 and 100 people, restrictions of all social gatherings (i.e., upper limit is lower than ten people), closure of restaurants and entertainment venues, closure of all non-essential shops, and nationwide lockdown (see Additional file 1: Table S1 for detailed definitions). Using the dates of decision/implementation, time-to-decision/implementation of measures was recorded using a global pandemic milestone − 11/3/2020- the date of the World Health Organization (WHO) declaration of pandemic, and a country-level epidemic milestone- the date 100 local cases were reached.

Data regarding the mitigation measures and outcomes were collected from several sources and cross referenced. We used official government websites, the Oxford COVID-19 Government Response Tracker,[5] the WHO European Observatory’s COVID-19 Health System Response Monitor,[4] the Health Intervention Tracking for COVID-19 (HIT-COVID) database [23], and the John’s Hopkins University Policy Tracker [24]. When data could not be found using these sources, we resorted to reputable news sources.

Infection and mortality data were collected from OWID, and for Hong Kong from the Ministry of Health database [25, 26]. For each country that had implemented a PHM, COVID-related mortality per million population at 60 days from the date of implementation was recorded. As the distribution of mortality per million skewed to the right, we performed a log transformation for these data.

For federal countries which had different PHMs implemented in different regions, we used the data available from the most populated state/province. These were Ontario, Canada; California, USA; New South Wales, Australia; and Sao Paulo, Brazil. Other federal countries in our study population implemented a centralized-national response to the first wave of the pandemic.

**Fig. 1:**
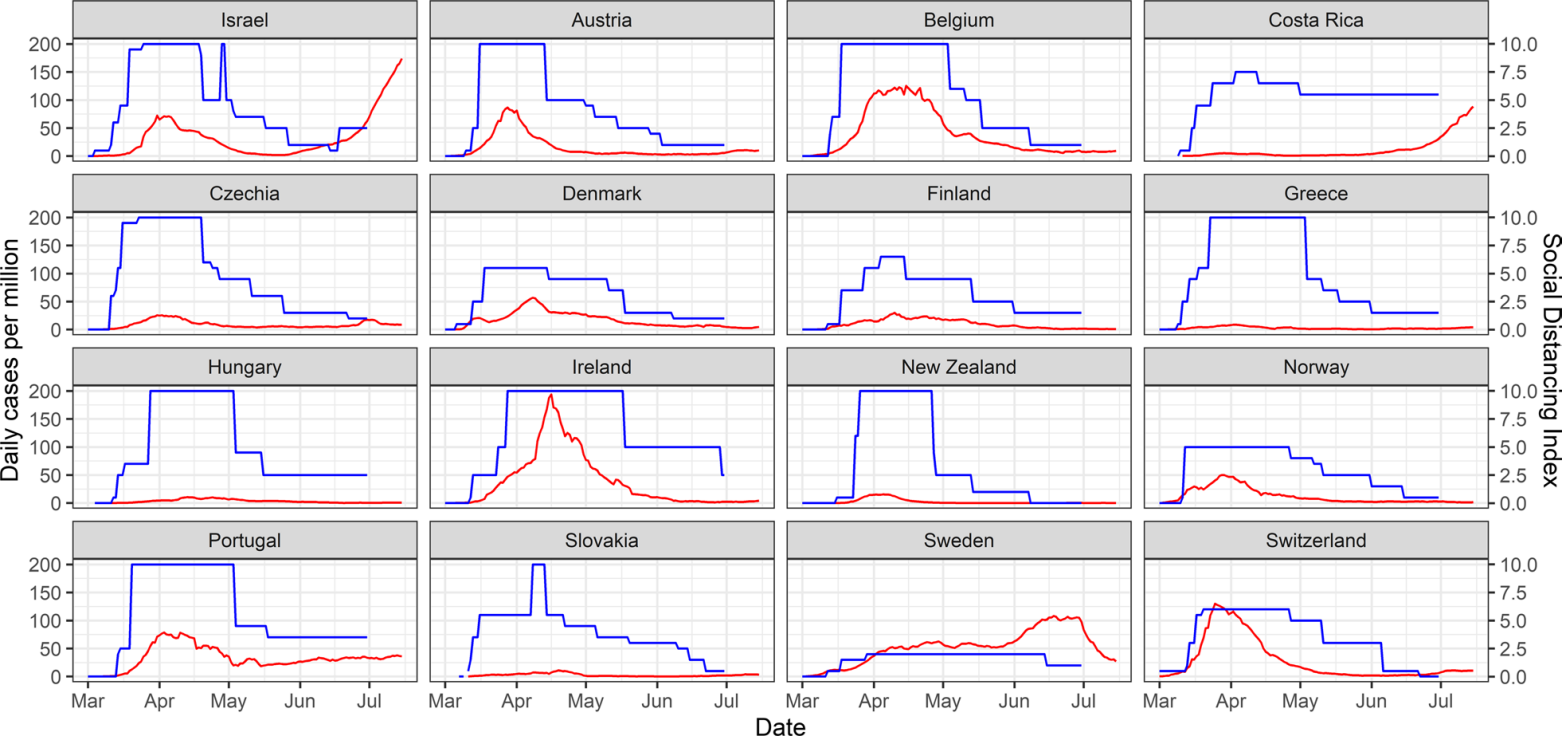
Social distancing index^a^ and new daily cases per million 01/3/2020-15/7/2020^b^. Blue line- social distancing index; red line- new daily cases per million. ^a^Components of the social distancing index: Movement restrictions: partial (night-time curfew/local quarantine) =2 points, nationwide lockdown=4 points. Social gatherings: limit of>100=0.5 points, limit of 50–100=1 point, limit of 10–49=1.5 points, limit of <10=2 points. Business closures: restaurants and entertainment venues=1 point, non-essential business=2 points. School closures: some but not all grades/schools=1 point, K-12 in entire country=2 points. ^b^Social distancing index was collected until 30/6/2020. Daily cases per million are presented until 15/7/2021 in order to present infection trends until two weeks later.

We constructed a “social distancing index” based on the four main components of social distancing measures implemented in Israel and ranges from 0 to 10 (complete scoring method is presented in Fig. 1). This index was not used in our statistical analysis, but only as a tool for visual comparison of the integrative PHMs taken by different countries, as the validity of the index was not fully established.

### Statistical analysis

Descriptive statistics include the timing of the ten PHMs for each county, measured according to time from the WHO “pandemic” declaration and as time from 100 confirmed COVID-19 cases in that particular country.

We graphically compared the social distancing index over time between 16 OECD countries with a similar population to that of Israel (4–12 million inhabitants). To compare the extent and speed of response between nations, we examined peak social distancing score and time-to peak score or nationwide lockdown (whichever came first) from initial social distancing measures.

All univariate analyses of association were performed twice- once for the entire study population and a sub-group analysis for OECD nations only. Pearson’s chi-square test and Fisher’s Exact test were used to assess the association of categorical variables. For comparison of means we used the independent t-test or analysis of variance (ANOVA) and the nonparametric Mann Whitney or Kruskal Wallis tests when the normality assumption was not met. To assess the association between continuous variables, we used linear regression models. For univariate analyses involving time-to-measure as a continuous variable, only countries that implemented the measures were included. Kaplan-Meier estimates were used for assessing survival curves and analyses were performed using the log-rank test. For these analyses the event was defined as the day of decision of each PHM. Time-to-event in days was measured for each country from the date that 100 local confirmed cases were reported. Time-to-event variables were corrected to positive numbers for purpose of the survival analyses. Countries that did not implement the measure were censored at 30/06/2020.

We adjusted the level of significance of *p*-values to account for multiple comparisons, according to the stringency of measures enacted. For comparisons regarding complete lockdown, a 2-sided *p*-value of 0.05 was considered significant. For non-essential business and primary school closures, given that these measures carry broad social and economic implications we considered a 2-sided *p*-value of 0.025 significant. For the remaining measures, a 2-sided *p*-value of 0.0071 was considered significant after using the Bonferroni correction.

Cox proportional hazards regression analyses were performed to assess factors related to time-to-decision for each of the ten measures. Event definition, event censoring and time-to-event-variables were defined as described above. Variables entered into the model were the pre-pandemic characteristics found to be associated with the measure in the univariate analyses mentioned above with a statistical significance of 0.2 or lower. These variables were entered into each regression model in their dichotomous form. The Schoenfeld test was used to verify the proportional hazards assumption. We controlled for potential confounding by geographic location by entering the “geography” variable, defined as Asia, Europe or Other, into all models. Hazard ratios were reported with a 95% confidence interval (CI), and adjusted levels of significance were dependent on the measure assessed, as specified above. Analyses were performed using a two-step approach: (1) “enter” method for geography; (2) “stepwise” forward likelihood ratio method for all other potential variables. For the latter step, entry was set at *p* = 0.05 and removal at P = of 0.10.

To assess the association between the timing of social distancing measures and COVID-19 related mortality, we performed a linear regression between the time-to-implementation of measures from the date of confirmation of 100 local cases to the log of total COVID-19-attributed deaths per million population at 60 days from implementation of the considered measure, while controlling for geographic location.

Regarding power, we based our calculation on a two-group comparison of the median time-to-measure-implementation each including 18 counties (36 total OECD at the time the study was formulated), assuming a standard deviation (SD) of 5 days (based on pilot data), and an α of 0.05- yielding a power of 83.15% to detect a difference of 5 days. Statistical analyses were performed in SPSS software for Windows version 25 (IBM Corp., Armonk, N.Y., USA); WinPEPI (PEPI-for-Windows) software version 11.65 (copyright J.H. Abramson, Aug 23, 2016); and R version 4.1.1 (R Core Development Team 2021).

## Results

### Pre-pandemic characteristics

Among the 50 countries assessed, Israel ranked highly (6th) in education, slightly above average in economic strength (18th), and below the OECD mean and median in democracy (25th), healthcare reserve (26th) and trust (35th) (Table 1, Additional file 1: Table S2).

The OECD countries as a group had a statistically significant higher score in all pre-pandemic characteristics, when compared to the twelve non-OECD countries (Table 1). There were several statistically significant differences in mean scores of pre-pandemic characteristics between countries from different geographic areas (Additional file 1: table S3).

**Table 1:** Pre-pandemic characteristics in study countries, Israel, OECD, and non-OECD countries *OECD vs. non-OECD countries

Pre-pandemic characteristic	All countries (n = 50)		Israel		OECD ^a^ (n = 38)		Non-OECD ^b^ (n = 12)		*p*-value*
	Mean (SD)	Median (IQR)	Score	Rank- among all countries (50)/OECD (38)	Mean (SD)	Median (IQR)	Mean (SD)	Median (IQR)	
EIU democracy index ^c12^	7.52 (1.62)	7.80 (6.89–8.71)	7.86	25 / 25	8.09 (1.11)	8.06 (7.50–9.05)	5.72 (1.71)	6.17 (3.66–6.99)	< 0.001
GDP per capita (USD) ^d13^	33,697 (25,242)	27,676 (12,075–49,136)	43,641	18 / 16	39,444 (23,951)	40,370 (19,424-51,820)	15,498 (20,815)	8262 (1888–21,652)	0.001
Educational attainment^e14^	31.18 (14.27)	30.60 (19.80–40.30)	47.10	6 / 5	34.78 (12.23)	34.10 (25.73–43.08)	19.80 (14.75)	14.65 (10.73–26.83)	0.001
Trust (percentage) ^f,17^	32.98 (17.78)	30.95 (21.18–41.08)	22.90	35 / 29	36.10 (18.38)	33.70 (23.40-49.15)	22.48 (10.61)	22.90 (16.6–30.80)	0.024
Hospital beds per 1,000 population ^15,16^	4.05 (2.57)	3.06 (2.51–5.61)	2.98	27 / 22	4.35 (2.67)	3.31 (2.60–5.75)	3.11 (2.05)	2.30 (1.6–4.98)	0.049

^a^OECD countries included: Australia, Austria, Belgium, Canada, Chile, Colombia, Costa Rica, Czech Republic, Denmark, Estonia, Finland, France, Germany, Greece, Hungary, Iceland, Ireland, Israel, Italy, Japan, Latvia, Lithuania, Luxembourg, Mexico, Netherlands, New Zealand, Norway, Poland, Portugal, Slovakia, Slovenia, South Korea, Spain, Sweden, Switzerland, Turkey, United Kingdom, United States

^b^Non-OECD countries included: Argentina, Brazil, Egypt, Hong Kong, India, Kenya, Russia, Rwanda, Singapore, South Africa, Taiwan, Thailand.

^c^EIU democracy index: 0–4=authoritarian regime; 4–6=hybrid regime; 6–8= flawed democracy; 8–10= full democracy

^d^GDP per capita adjusted per purchasing power parity in USD

^e^Educational attainment: percentage of population above the age of 25 with upper secondary education

^f^Trust: percent of people agreeing with the phrase "most people can be trusted". n=48/37 no available score for Costa Rica and Kenya

### Comparison of mitigation measures instituted in Israel and other countries

**Fig. 2:**
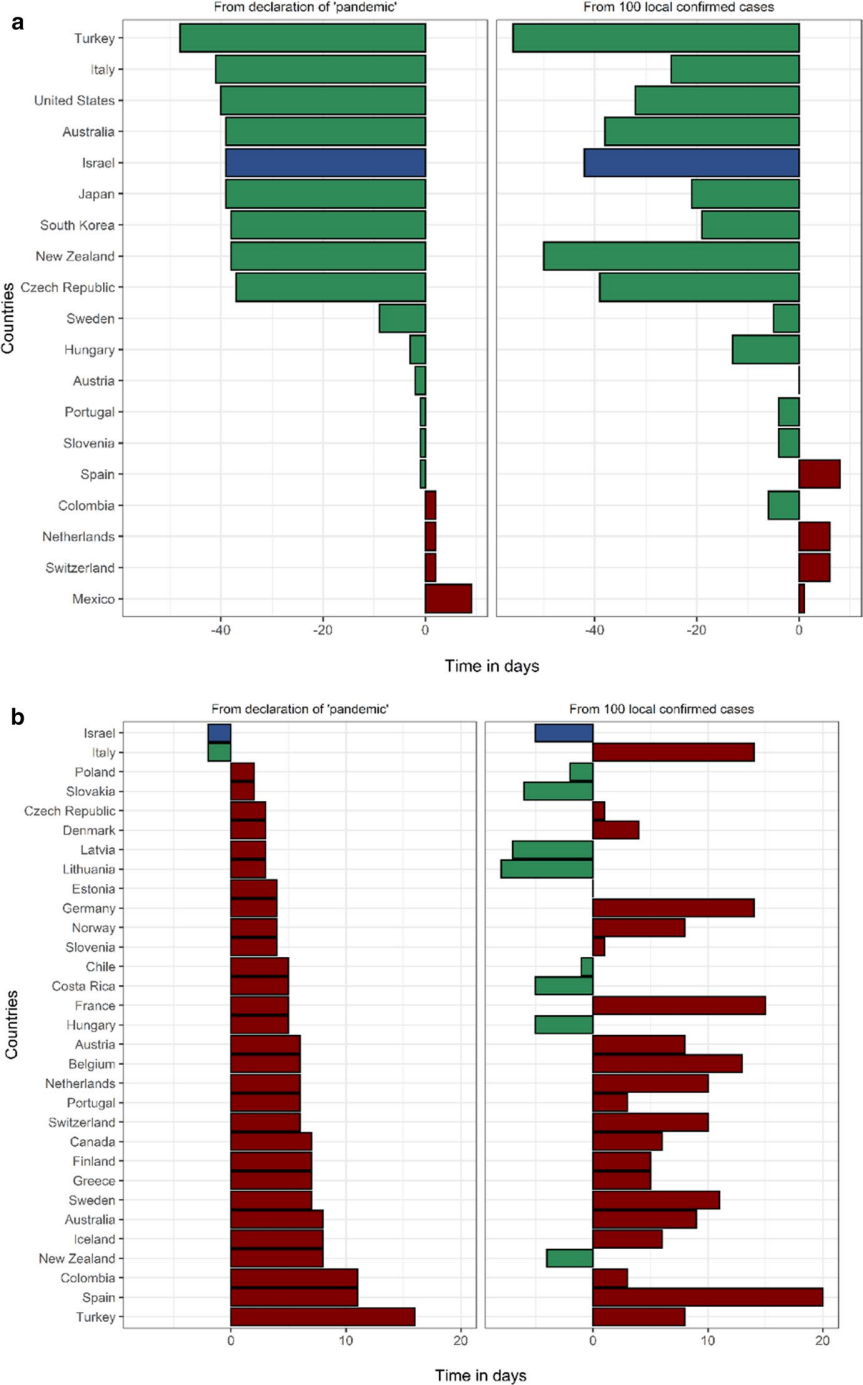

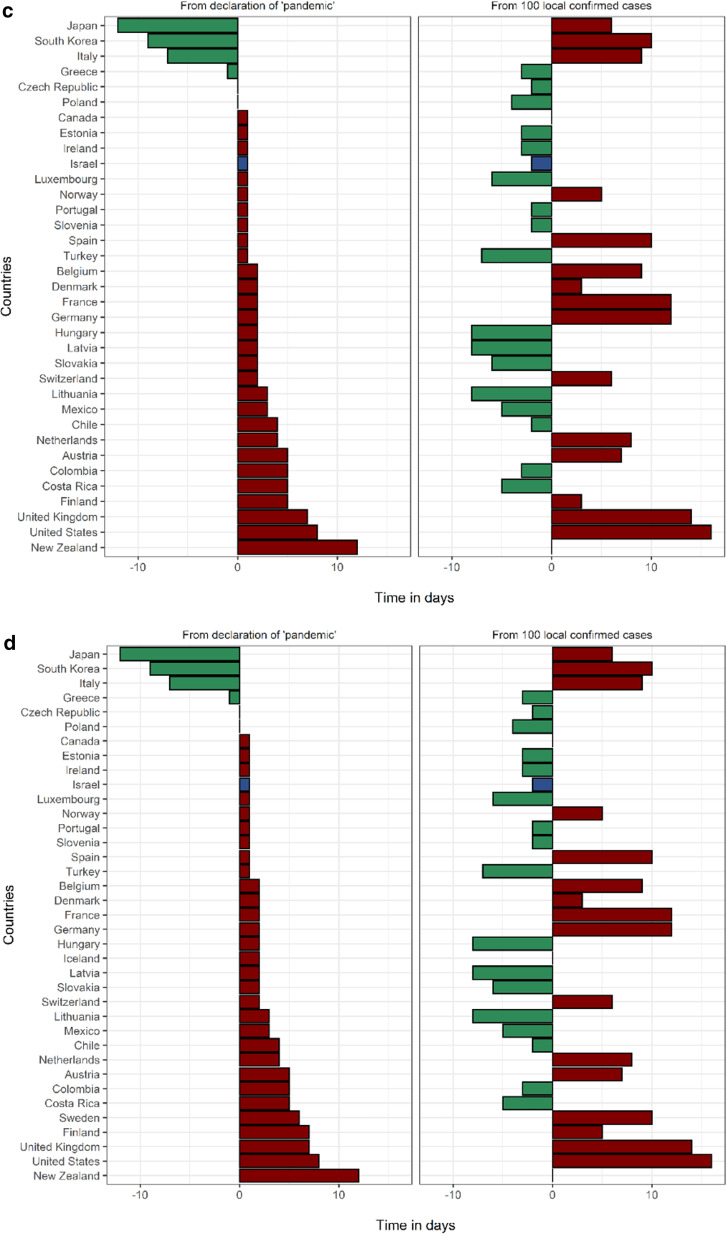

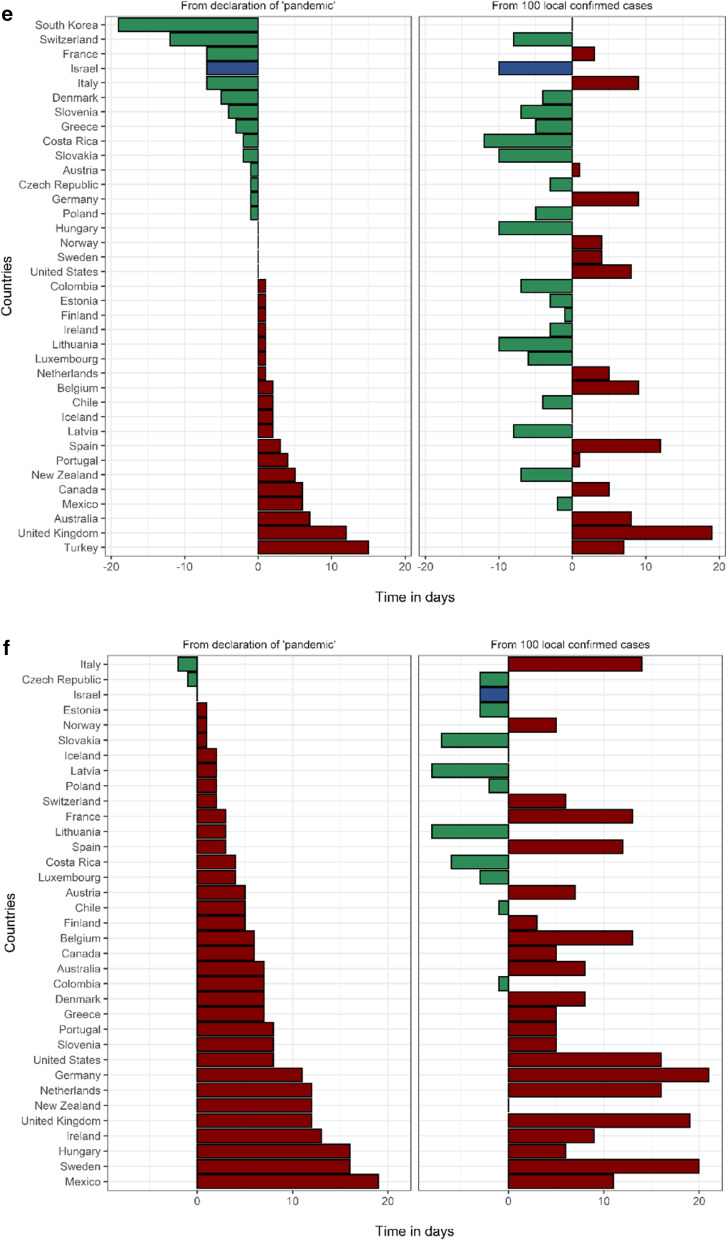

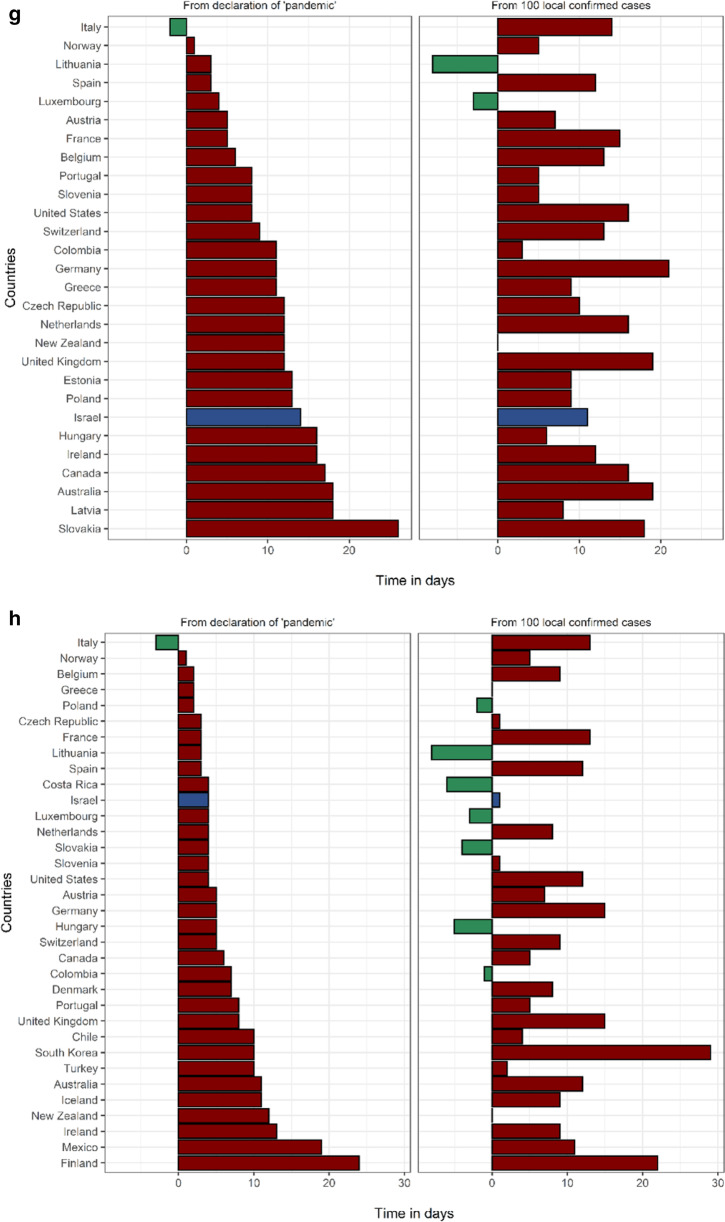

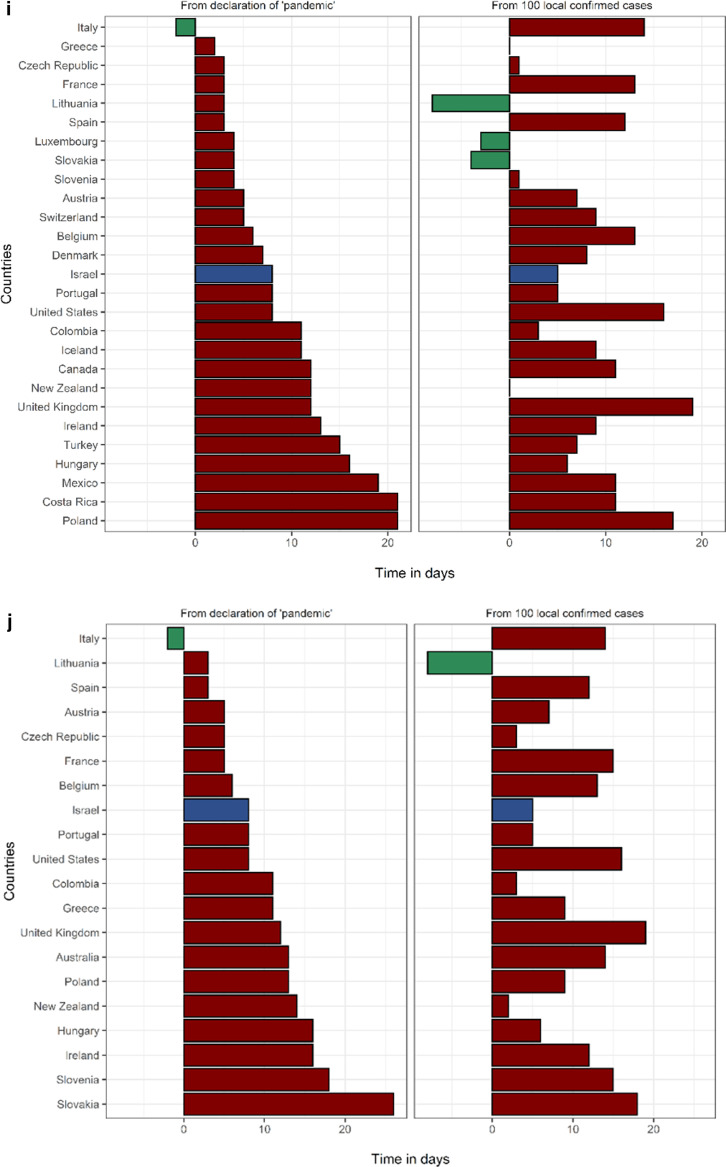
Timing of public health measures in OECD from "pandemic" (left) and from 100 cases. **a** Timing of selective entry restrictions among OECD countries. Countries that did not implement selective entry restrictions: Belgium, Canada, Chile, Costa Rica, Denmark, Estonia, Finland, France, Germany, Greece, Iceland, Ireland, Latvia, Lithuania, Luxembourg, Norway, Poland, Slovakia, United Kingdom. **b** Timing of complete border closures among OECD countries. Countries that did not implement complete border closure: Ireland, Japan, South Korea, Luxembourg, Mexico, United Kingdom, United States. **c** Timing of primary school closures among OECD countries. Countries that did not implement primary school closures: Australia, Iceland, Sweden. **d **Timing of secondary school closures among OECD countries. Countries that did not implement secondary school closures: Australia, Iceland, Sweden. **e ** Timing of cancellation of mass events among OECD countries. Countries that did not implement cancellation of mass events: Japan. **f** Timing of limitation of social gatherings to under 100 people among OECD countries. Countries that did not implement limitation of social gatherings to under 100 people: Japan, South Korea, Turkey. **g **Timing of limitation of social gatherings to under ten people among OECD countries. Countries that did not implement limitation of social gatherings to under ten people: Chile, Costa Rica, Denmark, Finland, Iceland, Japan, South Korea, Mexico, Sweden, Turkey. **h **Timing of closures of restaurants and entertainment venues among OECD countries. Countries that did not implement closure of restaurants and entertainment venues: Japan, Latvia, Sweden. **i** Timing of closures of non-essential businesses among OECD countries. Countries that did not implement closure of non-essential businesses: Australia, Chile, Estonia, Finland, Germany, Japan, South Korea, Latvia, Netherlands, Norway, Sweden. **j **Timing of nationwide lockdown among OECD countries. Countries that did not implement a nationwide lockdown: Canada, Chile, Costa Rica, Denmark, Estonia, Finland, Germany, Iceland, Japan, South Korea, Latvia, Luxembourg, Mexico, Netherlands, Norway, Sweden, Switzerland, Turkey

In Israel, the first measure taken to prevent the importation of COVID-19 cases was an entry restriction imposed on travelers from China on 1/2/2020. Israel was the 4th country in the OECD to announce selective entry restrictions, following Turkey, Italy and the USA. This measure came 42 days before 100 local cases were recorded in Israel, which ranks 3rd in the OECD in this metric (Fig. 2, Additional file 1: Table S4). The first measure of forced social distancing by the Israeli government was the cancellation of mass events with ≥ 5000 participants, on 4/3/2020, one week prior to the “pandemic” declaration by the WHO and ten days before Israel reached 100 local cases (tied for 2nd in the OECD). The 5000-person limit was lowered to 100 people one week later, on 11/3/2020, and was quickly followed by complete border closure to all foreigners and the closing of all educational facilities on 12/3/2020. On 15/3/2020 restaurants and entertainment establishments were shut down and on 19/3/2020 a nationwide lockdown was announced, ordering the closure of all non-essential shops and restricting people from leaving their homes with the exception of pre-defined essential activities such as grocery shopping, medical needs and work that cannot be performed remotely (under limitations). Fifteen days lapsed from the first PHM to the implementation of a nationwide lockdown. It is worth mentioning that on 9/3/2020 Israel celebrated Purim, a Jewish holiday characterized by large gatherings and parties. During the holiday weekend the only social distancing limitation in place was the prohibition of mass events with 5,000 or more people. There are data to suggest that the Purim celebrations facilitated the exponential growth of the epidemic in Israel, increasing the effective reproduction rate from 0.69 to 4.34 [27].

A comparison of the “social distancing index” in the 16 OECD countries with a similar population to Israel’s is presented in Fig. 1. The time-to-lockdown in Israel (15 days) was slightly longer than the mean time from 1st measure to peak social distancing among the 16 countries (13.7 days). The mean peak score on the social distancing index was 8.28 and the median score 10.0, with countries like Austria, Belgium, Czech Republic and Greece reaching a score of 10 like Israel. Meanwhile, Denmark, Finland, Norway and Switzerland all reached a peak score of 5–6.5, mainly due to lack of, or relatively lenient, movement restrictions in these countries. Sweden was an outlier with a peak score of two.

Only 15 of the 50 countries began implementing forced social distancing measures (cancellation of mass events) before the declaration of a state of pandemic by the WHO on 11/3/2020, and only three of these countries had done so more than a week prior to the pandemic declaration (Hong Kong, South Korea and Switzerland). Israel was the fourth country to implement forced social distancing measures (in the form of cancellation of mass events on 4/3/2020).

Israel ranked in the upper third of the OECD for timing for eight of the ten PHMs compared, when assessed as time-from- 100-cases and as time- from- “pandemic” (Fig. 2). Specifically, Israel ranked 2nd /3rd in cancellation of mass events, 4th /1st in complete border closures, 5th/3rd in limitations on gatherings of 100 people and 5th/8th in nationwide lockdown, when measured as days from 100 cases and days from “pandemic”, respectively. Timing of each measure among the entire study population is presented in Additional file 1: Table S4.

### Factors associated with measures implemented and their timing

#### Measure implementation

There was no statistically significant difference between OECD and non-OECD countries in our study in the proportion of countries implementing specific measures (Additional file 1: Table S5). Level of democracy, GDP per capita, trust and number of hospital beds per 1000 residents were not found to be associated with the implementation of any measure in the categorical analysis (Additional file 1: Table S6). A low education level was borderline associated with higher odds of implementing a nationwide lockdown (OR 3.27, 95% 95% CI = 0.99–10.98, *p* = 0.045) (Additional file 1: Table S6).

The implementation of several measures was almost ubiquitous across the study countries. Border closures, primary and secondary school closures and cancellation of mass events were implemented by more than 90% of the countries, while restaurant closure and limitations on social gatherings below 100 people were implemented by more than 85%. Implementation of these measures (yes/no) was not influenced by pre-pandemic characteristics. In contrast, a nationwide lockdown and selective border restrictions were implemented by only half of the study population (25 countries).

#### Time to implementation of measures

Several pre-pandemic characteristics were found to be associated with the timing of measures. A high GDP per capita and a high level of trust were associated with a longer median time-to-implementation of six and seven of the ten measures investigated, respectively, excluding countries that did not implement the measures in question (Table 2). These included school closures, border closure, restrictions on social gatherings, and business closures. In the OECD analysis similar associations were observed. Democracy level was associated with a longer median time-to-closure of both primary and secondary schools in the all-country analysis (Table 2). Education level and number of hospital beds per capita were not associated with median time-to-implementation of any of the measures investigated.

**Table 2:** Association of Democracy Index Scores, GDP per capita and Trust levels with median time-to-measure implementation

Measure/Pre-pandemic characteristic	High democracy level^a^ (n = 19*)		Low democracy level^a^ (n = 31* including Israel)		*p* value**	High GDP^b^(n = 21* including Israel)		Low GDP (n = 29*)		*p* value**	High trust index^c^ (n = 20*)		Low trust index (n = 28* including Israel)		*p*-value**
	# of countries	Days (median [IQR])	# of countries	Days (median [IQR])		# of countries	Days (median [IQR])	# of countries	Days (median [IQR])		# of countries	Days (median [IQR])	# of countries	Days (median [IQR])	
Primary school closure	16	4 [(− 1.5)–9.5]	31	− 3 [(− 7)–1]	**0.012**	18	5.5 [(− 0.5)–12]	29	− 3 [(− 6.5)–(− 0.5)]	**0.002**	17	6 [0–11]	28	− 2.5 [(− 6)–0.25]	**0.001**
Secondary schools	18	5 [(− 0.5)–10]	31	− 3 [(− 7)–1]	**0.006**	20	5.5 [0–11.5]	29	− 3 [(− 6.5)–(− 0.5)]	**0.001**	19	6 [0–10]	28	− 2.5 [(− 6)–0.25]	**0.001**
Selective entry restrictions	7	0 [(− 38)–6]	18	− 28.5 [(− 41.25)–(− 5.5)]	0.064	10	− 34.5 [(− 39)–1.5]	15	− 19 [(− 41)–(− 4)]	1.000	10	− 18.5 [(− 38)–6]	15	− 21 [(− 42)−(− 4)]	0.261
Border closure	16	8 [4.25–10.75]	27	0 [(−5)−8]	0.034	17	9 [5.5–13.5]	26	− 0.5 [(− 5)–3.5]	**0.001**	17	9 [5.5–13.5]	24	− 0.5 [(− 4.75)–4.5]	**0.001**
Cancellation of mass events	19	1 [(− 4)–5]	30	−3 [(− 8.5)–6.25]	0.192	21	3 [(− 5)–8]	28	− 3.5 [(− 8)–4]	0.091	20	4 [(− 3)–8.75]	27	− 4 [(− 8)–3]	0.022
Social gatherings limited to less than 100	19	7 [0–13]	25	5 [(− 3)-10.5]	0.117	21	8 [4–17.5]	23	− 1 [(− 6)–7]	**0.001**	20	8.5 [5–18.5]	22	− 1 [(− 3)–7]	**0.001**
Social gatherings limited to less than 10	13	13 [6–17.5]	23	8 [3–13]	0.115	17	15 [9–19]	19	7 [(− 1)–9]	**0.002**	16	14.5 [9.75–19]	19	7 [(− 1)–10]	**0.001**
Restaurants closed	18	8.5 [4.75–12.25]	25	2 [(− 1)–9.5]	0.082	19	9 [5–13]	24	1.5 [(− 1.75)–7.75]	**0.005**	17	9 [7.5–13.5]	25	1 [(− 1.5)–7.5]	**0.001**
Non-essential businesses closed	12	9 [7.25–11.75]	22	5.5 [(− 0.25)–11.5]	0.118	14	9 [(6.5–13.75)]	20	5.5 [(− 0.75)–10]	0.027	12	10 [8.25–15.25]	21	5 [(− 1)–7]	**0.002**
Lockdown	7	12 [7–15]	18	6.5 [2–13.25]	0.141	9	13 [6–15.5]	16	6.5 [0–11.25]	0.074	8	12.5 [8.25–15.5]	17	6 [1–11.5]	0.066

*Only countries who implemented the measures were included in the analysis

**p-values obtained using the Mann-Whitney test. *p*-values in bold are considered significant after adjusting for multiple comparisons

^a^High democracy level > 8.055; low democracy level ≤ 8.055

^b^High GDP per capita > 40,370.4 USD; low GDP per capita ≤ 40,370.4 USD

^c^High trust index > 33.70; low trust index ≤ 33.70

**Fig. 3:**
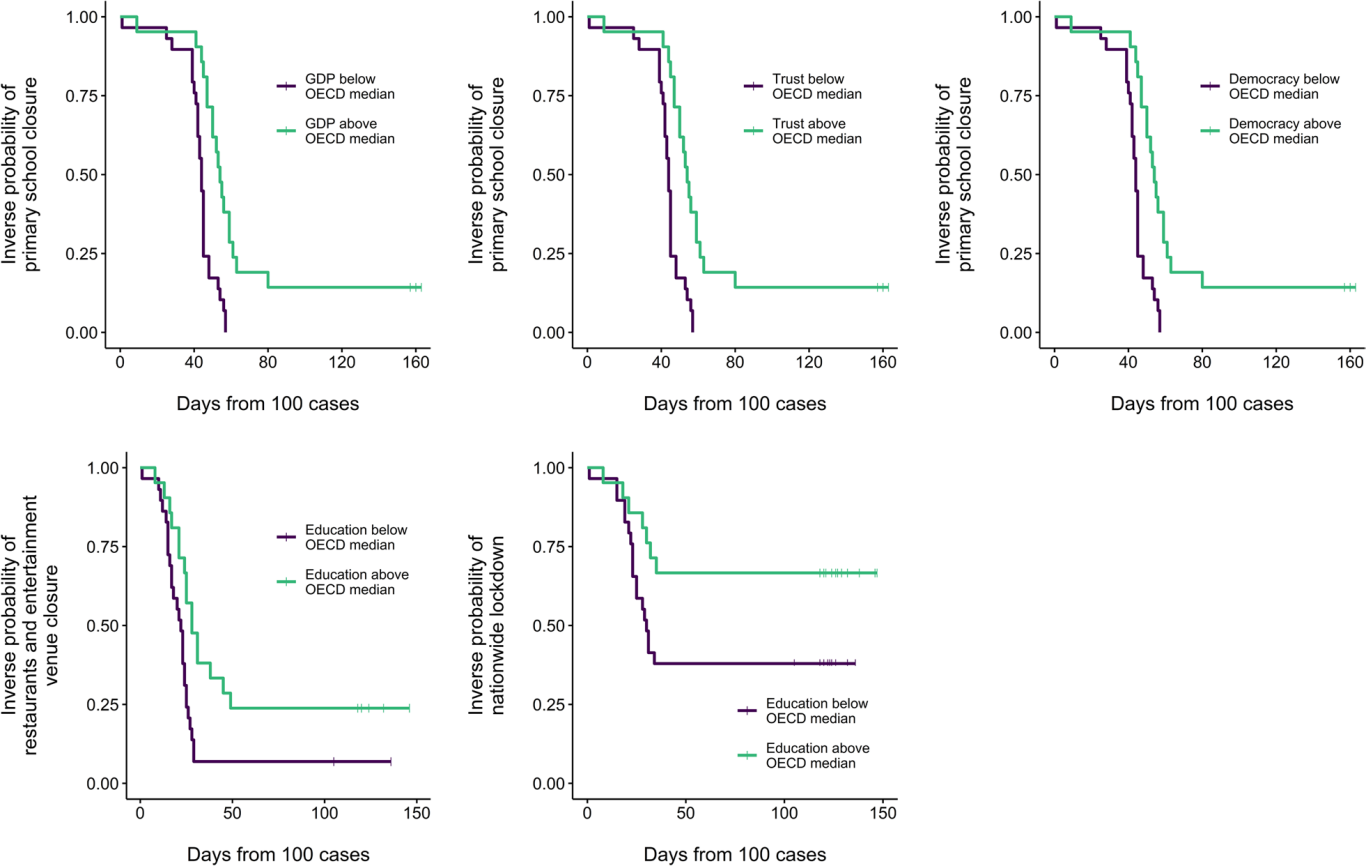
Kaplan–Meier curves and log rank tests comparing: (1) time to implementation of primary school closures between countries with high vs low GDP per capita (**a**), democracy (**b**) and trust (**c**); and time to restaurant and entertainment venue closure (**d**), and time to nationwide lockdown (**e**) between countries with high vs low education level.

In the Kaplan-Meier survival analysis, a high level of education was associated with a lower likelihood of a nationwide lockdown (log rank test *p*-value = 0.043) and restaurants and entertainment venues closure (*p*-value = 0.007) (Fig. 3). High levels of GDP (*p*-value < 0.001), Democracy (*p*-value = 0.004) and Trust (*p*-value < 0.001) were all associated with a lower likelihood of implementing school closures (Fig.3). Other measures showed a trend towards association with pre-pandemic characteristics, though these associations did not meet the pre-specified adjusted significance levels.

### Multivariate Cox regression analysis for implementation of PHMs

In a Cox model performed using the forward stepwise method while controlling for geographic location, only democracy level predicted the implementation of a nationwide lockdown, with an inverse association between democracy level and hazard of implementing a nationwide lockdown (HR = 0.348, 95% CI 0.139–0.875, *p*-value = 0.025) (Additional file 1: Table 7). Similarly, a high GDP level was associated with reduced risk of closing both primary schools (HR = 0.25, 95% CI = 0.12–0.51, *p*-value < 0.001) and secondary schools (HR = 0.28, 95% CI = 0.14–0.55, *p*-value < 0.001), while controlling for geographic location (Additional file 1: Table S7); and a high education level with reduced risk of closure of restaurant and entertainment venues (HR = 0.31, 95% CI = 0.18–0.72, *p*-value = 0.004), while controlling for geographic location (Additional file 1: Table S7). Other associations between pre-pandemic characteristics and risk of implementation of measures were observed but did not meet the adjusted alpha levels for significance.

### Association of timing of PHMs with COVID-19-related mortality

In linear regression, controlling for geographic location, the timing of each measure from 100 local cases was correlated with COVID-related mortality (as the log of deaths per million) at 60 days from implementation of the measure (Table 3).

A post-hoc analysis comparing mortality at 60 days from 100 local cases between countries that did implement a lockdown and selective entry restrictions (two measures that were implemented by exactly half of the countries in our study) to those who did not, showed no statistically significant differences between the groups.

In a linear regression model, controlling for geography, the interval between pandemic declaration and the date where 100 local cases were recorded was associated with mortality at 60 days from 100 local cases (entered as log of deaths per million) (model R = 0.755, *p* < 0.001, adjusted R square = 0.543 standardized beta = 0.400, *p*-value 0.001); meaning the earlier a country reached 100 cases, the higher the 60-day mortality was.

Due to multicollinearity, the above measures found to be associated with mortality could not be entered into a multivariate linear regression model.

**Table 3:** Association between timing of measures^a^ and COVID-19 related mortality^b^, controlling for geographic location

Measure	Adjusted model R^2^*	Standardized beta for measure	Measure *p*-value
Primary school closure	0.531	0.448	< 0.001
Secondary schools	0.539	0.488	< 0.001
Selective entry restrictions	0.789	0.444	0.003
Border closure	0.533	0.466	< 0.001
Cancellation of mass events	0.541	0.455	< 0.001
Social gatherings limited to less than 100	0.555	0.512	< 0.001
Social gatherings limited to less than 10	0.444	0.434	0.004
Restaurants closed	0.386	0.404	0.004
Non-essential businesses closed	0.400	0.507	0.001
Lockdown	0.470	0.498	0.004

*All models were significant at a *p*-value < 0.005

^a^In days from 100 local cases

^b^As the log of number of cumulative COVID-19 related deaths per million population at 60 days after the implementation of each measure

## Discussion

In the face of the COVID-19 pandemic, countries adopted different initial approaches in attempt to mitigate and contain the spread of the virus, some measures being universally adopted while others showing substantial variability in implementation. For example, primary school closure was adopted by 47 of the 50 countries in our study, while nationwide lockdowns were adopted by only half. In addition to the differences in measures implemented, countries varied in the speed of their implementation.

Regarding time-to-implementation of measures, our study revealed that a high education level was associated with decreased risk of implementing a nationwide lockdown and closure of restaurants and entertainment venues. This may imply that governments in countries with highly educated populations tend to trust their citizens to follow social distancing guidelines, and therefore not feel the need to enforce a strict lockdown. Israel was somewhat of an outlier in this relationship, implementing a nationwide lockdown despite being ranked 6th in education level among the study countries. A similar inverse relationship was seen between levels of trust, GDP per capita and democracy and school closures.

When comparing and analyzing the timing of measures, several pre-pandemic characteristics were correlated with specific measures on univariate analyses. In all of these associations we observed that a higher level on these scales was associated with a longer time-to-measure (from confirmed 100 cases). This could be explained by the large share of European countries in our study population (26/50), whose mean pre-pandemic characteristic scores were higher than those in most other regions. However, even after controlling for geographic location in the multivariate regression, this observed trend persisted. Having a high democracy score was associated with a lower tendency to implement lockdowns, even when controlling for geographic location. This implies that countries with high democracy scores may be less inclined to implement lockdowns or only do so as a last resort. Israel was relatively quick in this regard, being the 8th in the OECD to do so (19/3/2020), and the 5th when measured in days from 100 local cases. Similar to democracy score, a high GDP was associated with a lower tendency for school closures and a high level of education with decreased probability of closure of restaurants and entertainment venues. Interestingly, the number of hospital beds per capita did not seem to play a role in influencing public health decision making in the first wave of the pandemic, although it is possible that other healthcare characteristics that were not assessed such as rates of physicians, intensive care unit beds, and ventilators had some influence on decision makers. Waitzberg et al. suggest in their review of the initial pandemic response in seven Mediterranean countries, including Israel, that these countries may have acted early in response to the first wave of the pandemic in part due to concerns related to healthcare capacity (all seven countries ranked below the European average in health expenditure and curative beds per capita) [28].

Many pre-pandemic characteristics mutually correlated. For this reason, they were entered individually into our final model in a stepwise fashion. All of these associations reflect the general trend in our study that “high-income” countries were less likely to implement social distancing measures early. Interestingly, Israel was early to implement most measures, despite ranking high in education and GDP.

Our findings differ from those of a pre-print study examining factors associated with school closures in the pandemic, which showed quicker implementation in more democratic countries [11]. This contradiction could be explained by methodological differences; Cronert et al. used a chronological timeline for their survival analysis with a reference point of a set date, while we used individual country timelines, with a reference point of the date of 100 local cases, although they did account for local caseload in their base probability [11]. It is also possible that the larger sample size included in their study (167 countries) revealed associations not seen in our study with a sample size of 50. Several other studies have looked into the question of determinants influencing public health decisions during the COVID-19 pandemic. Adolph et al. showed that in addition to state level caseload and number of neighboring states implementing measures, political partisanship had a larger effect on states’ decisions to implement social distancing measures in the USA [12]. They also showed that poorer states (with a lower GSP) were less likely to implement stricter measures. This differs from our study where “low-income” countries were more likely to implement measures and did so more rapidly. Comparison of these two studies is limited, since our study compared at a country level while Adolphe et al. compared at the US state level. In contrast with Adolph, Djulbegovich et al. found that political partisanship did not influence the decision to issue stay-at-home orders in the United States, but rather that the decision was influenced primarily by number of infections and deaths [8]. In a recent working paper, Gisselquist et al. analyze the relationship between aspects of state capacity and COVID-19-related health policy and outcomes [13]. Their initial analyses show an association between state capacity and timing of measures and an inverse association between measures of state capacity and the intensity of COVID-19 containment policy. These observations are similar to the trend observed in our study, as high-income countries tend to have higher state capacity, defined by the authors as the “ability to provide basic public services” [13]. However, when controlling for factors such as GDP per capita and geographic location, these associations were lost, and the only predictive measure found to be inversely associated on multivariate analysis with stringency of government response was “state legitimacy”. This finding is in line with our finding that high levels of trust were inversely associated with school closures [13].

The effectiveness of PHMs in containing the pandemic has been addressed in the recent literature [7, 9, 10, 29]. Medline et al. found that the peak of both infection and mortality occurred later in countries and regions that delayed the implementation of stay at home orders [7]. Auger et al. observed an association between school closures in US states and a decline in incidence of infections and mortality [9]. In contrast, Rice et al. came to the opposite conclusion in their modeling study in the UK, that while school closures reduced intensive care unit hospitalization in the short term, they actually increased overall mortality by prolonging the pandemic [30]. A recent meta-analysis reported an association between handwashing, mask wearing and physical distancing and reduced incidence of COVID-19, though they did not single out specific social distancing methods [10].

Israel was relatively quick to respond with initial PHMs, both on a local (time from 100 cases) and a global scale (time from “pandemic” declaration). However, as we have shown (Fig. 2), these two measurements do not always align. Hence, while two countries may have implemented a specific measure on the same day (eg. border closures in Israel and Italy), the stage of the local outbreak at the time was likely an important factor affecting the effectiveness of the measure. Earlier implementation of border closures, cancellation of mass events, limitations on social gatherings, school closures and business closures were all associated with lower 60-day mortality. There is clearly a correlation between earlier implementation of measures and reduced mortality, as expected, though without a clear indication of the relative effectiveness of specific measures. The time between 100 local cases and the date of “pandemic declaration” was also associated with mortality. This suggests that countries with lower local caseloads on 11/3/2020, had better outcomes. These findings put together suggest that perhaps more important than which measures were implemented and when, extent of local spread of the virus at the time the world started to take notice was the determining factor. This observation is also made by Waitzberg et al., who observed a “domino effect” of the outbreaks in Italy and Spain on neighboring countries [28]. The fact that the early timing of a relatively lenient social distancing measure such as cancellation of mass events was correlated with reduced mortality suggests that early implementation of any social distancing measures, rather than specific measures, affected this outcome. Conversely, this measure may have mitigated super-spreader events. Another example is the timing of selective entry restrictions, which was the earliest measure implemented. Among countries that implemented this early measure, earlier implementation was associated with lower mortality. Israel was one of the first to implement selective entry restrictions on travelers from China. In order to achieve rapid and effective infection control, a timely coordinated response is needed on an international level. This is heavily dependent on the sharing of information and international collaboration through organizations such as the WHO. With regards to the effectiveness of PHMs in infection control, it is important to state that throughout the pandemic PHMs were lifted and modified. It is beyond the scope of this study, which focuses on the initial response to the pandemic, to account for the impact of each specific measure on infections and deaths.

These observations could explain Israel’s relative success in the first wave of the pandemic. Alongside being among the first countries to implement several PHMs, Israel’s geographic distance from the early epicenters in China and Western Europe enabled these measures to be implemented early on the local timeline as well, while the virus was not yet widespread.

It is worth mentioning that the management of the pandemic in Israel was at the highest levels of government, with public health professionals serving solely in an advisory role. This trend was observed in other Mediterranean countries [28]. Conversely, Nordic countries relied extensively on the recommendations of public health agencies, leading to fewer formal restrictions in the first wave of the pandemic [31]. When the pandemic broke out, Israel was in the midst of a longstanding political crisis, having experienced three elections in less than a year. The third of these was held on 2/3/2020, one week after the first case of COVID-19 was identified in the country. The new government was sworn in on 17/5/2020, for the first two months, the pandemic was managed by a transitional government [32]. In a recent paper Maor et al. analyzed the management of the first wave of the COVID-19 pandemic in Israel from a policy perspective[32]. They suggest that the management constituted a deliberate disproportionate policy by Prime Minister Netanyahu both in rhetoric as well as in policy decisions.

Regarding the question of the necessity of a strict lockdown to control the COVID-19 pandemic, there are observational data from the Nordic and Asian states to support the notion that the first wave of the pandemic could be managed with relative “lenient” social distancing, yet the necessity for Israel’s to lockdown cannot be assessed directly using our methodology. Nonetheless, with regards to public health policy, our study shows that while Israel’s response was on the stringent end of the spectrum, it was not extraordinary.

This study aimed primarily to address factors influencing initial public health decision-making early in a pandemic, and to assess the effect of measures and their timing on mortality. The strict PHMs implemented early by Israel and other countries appeared to impact the short-term outcomes of infection related mortality, possibly at the expense of long-term outcomes related to measures such as school closures and economic slowing. The broader social, health and economic long-term effects of strict social distancing measures such as a lockdown and school closures were not considered in this study. There is however a growing body of literature that deals with these effects, and they should always be taken into account in the debate over the necessity and effectiveness of forced social distancing measures [33]. Arbel et al. calculated that the cost of the first lockdown in Israel was 36.4–38.6 billion new Israeli Shekels (NIS) and that the cost of a COVID-19 death prevented was above 36 million NIS. As a comparison, they calculated that the cost of a death prevented during the primary vaccination campaign was approximately 21 thousand NIS [34]. The tradeoff between democratic values and strict PHMs is an open question not answered by this study. While we have showed an inverse association between democracy level and readiness to implement PHMs, it would be interesting to further assess the association of factors such as democracy level and trust on the compliance of the population to PHMs, as well as the influence of strict PHMs implemented on subsequent levels of trust in government [35]. A review by Devine et al. found that levels of trust were associated with higher compliance to PHMs, at least early in the epidemic, although whether this was sustained in later phases is not addressed in the review [36].

Our study has several limitations. As an ecological study, it is at high risk for confounding. In addition, despite applying definitions and criteria, country-specific measures may differ in their details. Our indicators are limited in their ability to adequately represent each social and health care domain, although we utilized commonly used indicators, chosen based on expert opinion. The sample size was limited. In addition, we had to approximate and extrapolate data from states and provinces to a country level, which again increases the chance of misclassification and bias. Finally, this study covers only the early pandemic response, while country-level responses, have evolved over time. Furthermore, the availability of vaccines and the development of viral variants of SARS-CoV2 have certainly altered the landscape.

Our study has several strengths. We analyzed a broad range of PHMs in response to the pandemic, breaking down state level responses to several layers, thus enabling comparisons between-countries. In addition, we present a unique comparative view focusing on the steps taken in Israel as compared with other countries with similar populations. Finally, our study sought to address the unanswered question of why some countries react differently than others to a similar threat, by assessing how countries’ baseline characteristics are related to their initial pandemic response.

In summary, Israel’s response was relatively quick when compared to other OECD countries both in calendar date as well as on the local timeline. Countries that ranked higher in pre-pandemic socio-economic indices were less inclined to implement strict PHMs and did so later than countries that ranked lower. Finally, we did not identify a specific package of PHMs which proved superior in reducing 60-day mortality. Instead, our data suggest that timing of initiation of measures relative to the country-specific spread of disease was a more significant factor than the exact measures implemented. Further research on the long-term social impacts of initial pandemic response is warranted.

## Conclusion

We conclude that a country’s level of democracy, economic strength, and the educational level of its populace influence the speed and strictness of pandemic PHMs at the early stages before vaccines become available. A timely pandemic response is crucial and requires international coordination. It is unclear which measures were most effective in slowing COVID-19 progression and this should be the subject of further studies. COVID-19 will not be the last pandemic the world faces, and findings of this study can be brought to bear on future decision-making in the face of uncertainty.

## Supplementary Information

**Additional file 1. Table S1**: Ten public health measures used as dependent variables and their definitions. List of the ten PHMs defined and recorded for each country and their definition. **Table S2**: Pre-pandemic characteristics- rank and score for each study country. List of the 50 countries included in the study and their rank (among study countries) and score in each of the 5 pre-pandemic characteristics recorded. **Table S3**: Differences in mean pre-pandemic characteristics between countries from different geographic locations. Comparison of mean pre-pandemic characteristics between different geographic regions- Asia, Europe, Latin America, Africa, North Americana and Oceania. **Table S4**: Public health measures implemented by each country: time in days from 100 cases and time in days from 11/3/2020 (date of "pandemic" declaration by the WHO). List of the 50 study countries and the timing of implementation of each public health measure, presented as time from 100 cases and as time from 11/3/2020 (date of "pandemic" declaration by the WHO).**Table S5**: Comparison of rates and percentage of countries implementing each measure in the OECD nations versus non-OECD nations. Rates of implementation of each public health measure in entire study population, in OECD nations and in non-OECD nations. **Table S6**: Categorical association between implementation of measures and high/low level of: GDP, trust, Democracy Index and number of hospital beds per population. Rates of implementation of each public health measure compared between countries with above median levels of pre-pandemic characteristics to countries with below median levels of pre-pandemic characteristic, for each of the five pre-pandemic characteristics. **Table S7**: Full Cox proportional hazards model of: nationwide lockdown (7a), primary school closure (7b), secondary school closure (7c); and restaurant and entertainment venues closure (7d); by pre-pandemic characteristics and geographic location. Variables entered into Cox proportional hazards model of four public health measures: nationwide lockdown, primary school closure, secondary school closure, and restaurant and entertainment venues closure.

## Data Availability

The datasets generated and/or analyzed during the current study are available for download on the Israel National Institute for Health Policy Research **(**NIHP**)** website [37].

## Abbreviations

COVID-19: Coronavirus disease 2019
PHMs: Public health measures
OECD: Organisation for economic co-operation
EIU: Economist intelligence unit
GDP: Gross domestic product
WB: World Bank
OWID: Our world in data
WHO: World Health Organization
CI: Confidence interval
NIS: New Israeli Shekel
