# Supplementary material for: Timing, extent and outcomes of public health measures in the first wave of the COVID-19 pandemic in Israel and a comparative analysis by socioeconomic indices
Source: Isr J Health Policy Res. 2023 Jan 30;12:5. doi: 10.1186/s13584-022-00549-2 (PMC9885622; doi:10.1186/s13584-022-00549-2)
Supplement: Supplementary file 1 — Additional file 1. Table S1: Ten public health measures used as dependent variables and their definitions. List of the ten PHMs defined and recorded for each country and their definition. Table S2: Pre-pandemic characteristics- rank and score for each study country. List of the 50 countries included in the study and their rank (among study countries) and score in each of the 5 pre-pandemic characteristics recorded. Table S3: Differences in mean pre-pandemic characteristics between countries from different geographic locations. Comparison of mean pre-pandemic characteristics between different geographic regions- Asia, Europe, Latin America, Africa, North Americana and Oceania. Table S4: Public health measures implemented by each country: time in days from 100 cases and time in days from 11/3/2020 (date of "pandemic" declaration by the WHO). List of the 50 study countries and the timing of implementation of each public health measure, presented as time from 100 cases and as time from 11/3/2020 (date of "pandemic" declaration by the WHO).Table S5: Comparison of rates and percentage of countries implementing each measure in the OECD nations versus non-OECD nations. Rates of implementation of each public health measure in entire study population, in OECD nations and in non-OECD nations. Table S6: Categorical association between implementation of measures and high/low level of: GDP, trust, Democracy Index and number of hospital beds per population. Rates of implementation of each public health measure compared between countries with above median levels of pre-pandemic characteristics to countries with below median levels of pre-pandemic characteristic, for each of the five pre-pandemic characteristics. Table S7: Full Cox proportional hazards model of: nationwide lockdown (7a), primary school closure (7b), secondary school closure (7c); and restaurant and entertainment venues closure (7d); by pre-pandemic characteristics and geographic location. Variables entered into Cox [file 13584_2022_549_MOESM1_ESM.docx]

Supplementary table 1: Ten public health measures used as dependent variables and their definitions

| **Measure** | **Definition** |
| --- | --- |
| Closing of primary schools | Closure of most primary education systems (more than 50% of students sent home). |
| Closing of secondary education | Closure of most secondary education systems and universities (more than 50% of students sent home). |
| Selective entry restrictions | No entry allowed for foreigners arriving from specific countries but not complete border closure. |
| Complete border closures | Restriction of entry to all foreigners. For European Union nations defined as restriction on entry for non-EU citizens. |
| Cancellation of mass public events | Prohibition of public events. Defined as a maximum number of participants allowed above 100. |
| Limitations on social gatherings of 100 people or less | Prohibition of social gatherings with a maximum number of participants between 10 and 100. |
| Limitations on all social gatherings | Prohibition of social gatherings with a maximum number of participants below 10. |
| Restaurants and entertainment venues | Restriction on the operation of hospitality industry including restaurants, bars, cinemas. etc. (not including take away and curbside pick-ups). |
| Closure of non-essential businesses | Restriction on the operation of all shops and in-person business. Essential services exempted (e.g. food, medicine and certain social services. There may be variation between countries). |
| Lockdown | Restriction on movement of citizens outside a specified vicinity of their place of residence) except for essential needs such as exercise, food shopping and medical services. Ranging from strict stay-at-home orders to a restriction to leave one's neighborhood, borough or town. |

Supplementary table 2: Pre-pandemic characteristics- rank and score for each study country

| **Country** | **EIU Democracy Index** | | **World Bank- GDP per capita in USD** | | **World Bank- Educational attainment** | | **Hospital beds per 1,000 people** | | **World Value Survey- Trust** | |
| --- | --- | --- | --- | --- | --- | --- | --- | --- | --- | --- |
|  | **Rank** | **Score** | **Rank** | **Score** | **Rank** | **Score** | **Rank** | **Score** | **Rank** | **Score** |
| **Australia** | 9 | 9.09 | 9 | 54,907 | 5 | 48.30 | 20 | 3.84 | 10 | 48.5 |
| **Austria** | 15 | 8.29 | 12 | 50,277 | 27 | 29.40 | 4 | 7.27 | 9 | 49.8 |
| **Belgium** | 28 | 7.64 | 17 | 46,117 | 22 | 33.80 | 13 | 5.58 | 19 | 34.6 |
| **Canada** | 7 | 9.22 | 16 | 46,195 | 2 | 60.40 | 38 | 2.52 | 12 | 41.1 |
| **Chile** | 19 | 8.08 | 37 | 14,897 | 34 | 22.00 | 35 | 2.60 | 43 | 12.9 |
| **Colombia** | 36 | 7.13 | 45 | 6,432 | 36 | 21.30 | 44 | 1.71 | 48 | 4.5 |
| **Costa Rica** | 17 | 8.13 | 38 | 12,238 | 35 | 21.40 | 48 | 1.1 | N/A | N/A |
| **South Africa** | 34 | 7.24 | 49 | 1,120 | 43 | 15.20 | 33 | 2.8 | 33 | 23.5 |
| **Argentina** | 37 | 7.02 | 40 | 10,006 | 38 | 20.00 | 15 | 5.0 | 38 | 19.2 |
| **Czech Republic** | 27 | 7.69 | 30 | 23,102 | 37 | 20.20 | 7 | 6.62 | 37 | 21.1 |
| **Denmark** | 7 | 9.22 | 8 | 59,822 | 18 | 37.10 | 34 | 2.60 | 1 | 73.9 |
| **Estonia** | 24 | 7.90 | 28 | 23,660 | 13 | 40.30 | 37 | 2.57 | 20 | 33.9 |
| **Rwanda** | 48 | 3.16 | 50 | 802 | 50 | 4.10 | 46 | 1.6 | 41 | 16.6 |
| **Finland** | 5 | 9.25 | 14 | 48,686 | 19 | 36.20 | 21 | 3.61 | 3 | 68.4 |
| **France** | 18 | 8.12 | 21 | 40,494 | 26 | 30.20 | 10 | 5.91 | 29 | 26.3 |
| **Germany** | 13 | 8.68 | 15 | 46,259 | 20 | 35.60 | 3 | 8.00 | 11 | 43.4 |
| **Greece** | 33 | 7.43 | 31 | 19,583 | 32 | 26.70 | 19 | 4.20 | 45 | 8.4 |
| **India** | 38 | 6.90 | 47 | 2,104 | 48 | 10.00 | 50 | 0.5 | 40 | 16.7 |
| **Hungary** | 40 | 6.63 | 35 | 16,476 | 29 | 28.50 | 6 | 7.01 | 27 | 27.2 |
| **Iceland** | 2 | 9.58 | 5 | 66,945 | 25 | 31.00 | 32 | 2.83 | 5 | 62.3 |
| **Russia** | 49 | 3.11 | 39 | 11,585 | 45 | 14.10 | 5 | 7.1 | 34 | 22.9 |
| **Ireland** | 6 | 9.24 | 3 | 78,661 | 11 | 42.90 | 27 | 2.97 | 15 | 38.9 |
| **Israel** | **25** | **7.86** | **18** | **43,641** | **6** | **47.10** | **26** | **2.98** | **35** | **22.9** |
| **Italy** | 29 | 7.52 | 23 | 33,190 | 44 | 15.00 | 25 | 3.14 | 28 | 26.6 |
| **Japan** | 22 | 7.99 | 22 | 40,247 | 21 | 34.40 | 1 | 12.98 | 21 | 33.7 |
| **Brazil** | 39 | 6.86 | 43 | 8,717 | 41 | 16.50 | 41 | 2.2 | 47 | 6.5 |
| **Korea, South** | 21 | 8.00 | 24 | 31,762 | 12 | 40.30 | 2 | 12.43 | 22 | 32.9 |
| **Latvia** | 32 | 7.49 | 34 | 17,836 | 10 | 43.60 | 14 | 5.49 | 32 | 23.9 |
| **Egypt** | 50 | 3.06 | 46 | 3,020 | 46 | 13.00 | 45 | 1.6 | 46 | 7.3 |
| **Lithuania** | 30 | 7.50 | 32 | 19,456 | 4 | 54.60 | 9 | 6.43 | 23 | 31.7 |
| **Luxembourg** | 12 | 8.81 | 1 | 114,705 | 1 | 69.10 | 18 | 4.26 | 24 | 31.1 |
| **Mexico** | 43 | 6.09 | 41 | 9,863 | 42 | 16.40 | 49 | 0.98 | 44 | 10.5 |
| **Netherlands** | 11 | 9.01 | 10 | 52,448 | 23 | 33.30 | 24 | 3.17 | 6 | 58.5 |
| **New Zealand** | 4 | 9.26 | 20 | 42,084 | 7 | 46.10 | 36 | 2.57 | 8 | 56.6 |
| **Kenya** | 46 | 5.18 | 48 | 1,817 | 49 | 7.90 | 47 | 1.4 | N/A | N/A |
| **Norway** | 1 | 9.87 | 4 | 75,420 | 15 | 39.60 | 22 | 3.53 | 2 | 72.1 |
| **Poland** | 41 | 6.62 | 36 | 15,595 | 31 | 28.20 | 8 | 6.54 | 31 | 24.1 |
| **Portugal** | 20 | 8.03 | 29 | 23,145 | 39 | 19.20 | 23 | 3.45 | 39 | 17.2 |
| **Slovakia** | 35 | 7.17 | 33 | 19,329 | 33 | 22.80 | 12 | 5.70 | 36 | 21.4 |
| **Slovenia** | 30 | 7.50 | 26 | 25,739 | 30 | 28.20 | 17 | 4.43 | 30 | 25.3 |
| **Spain** | 15 | 8.29 | 25 | 29,614 | 24 | 31.20 | 28 | 2.97 | 13 | 41.0 |
| **Sweden** | 3 | 9.39 | 11 | 51,610 | 17 | 38.60 | 42 | 2.14 | 4 | 62.8 |
| **Switzerland** | 10 | 9.03 | 2 | 81,994 | 14 | 40.20 | 30 | 2.85 | 7 | 57.1 |
| **Turkey** | 47 | 4.09 | 42 | 9,043 | 40 | 19.10 | 31 | 2.85 | 42 | 14.0 |
| **United Kingdom** | 14 | 8.52 | 19 | 42,300 | 9 | 44.10 | 39 | 2.46 | 14 | 40.2 |
| **Hong Kong** | 44 | 6.02 | 13 | 48,756 | 28 | 29.10 | 16 | 4.9 | 18 | 36.4 |
| **Singapore** | 45 | 6.02 | 6 | 65,233 | 3 | 55.80 | 40 | 2.4 | 16 | 38.5 |
| **Taiwan** | 26 | 7.73 | 27 | 25,008 | 16 | 39 | 11 | 5.7 | 25 | 30.8 |
| **Thailand** | 42 | 6.32 | 44 | 7,808 | 47 | 12.90 | 43 | 2.1 | 26 | 28.9 |
| **United States** | 23 | 7.96 | 7 | 65,118 | 8 | 45.20 | 29 | 2.87 | 17 | 37.0 |

Supplementary table 3: Differences in mean pre-pandemic characteristics between countries from different geographic locations

|  | A | B | Mean Difference (A-B) | Lower | Upper | p-value* |
| --- | --- | --- | --- | --- | --- | --- |
| Democracy | Asia | Europe | -1.84 | -3.13 | -0.55 | **0.02** |
|  |  | Latin America | -0.81 | -2.61 | 0.98 | 1.00 |
|  |  | Africa | 1.74 | -0.31 | 3.8 | 1.00 |
|  |  | NA and Oceania | -2.48 | -4.53 | -0.43 | **0.04** |
|  | Europe | Latin America | 1.03 | -0.54 | 2.60 | 0.59 |
|  |  | Africa | 3.59 | 1.72 | 5.45 | **0.01** |
|  |  | NA and Oceania | -0.64 | -2.50 | 1.23 | 1.00 |
|  | Latin America | Africa | 2.56 | 0.32 | 4.80 | 1.00 |
|  |  | NA and Oceania | -1.66 | -3.90 | 0.58 | 0.26 |
|  | Africa | NA and Oceania | -4.22 | -6.68 | -1.77 | **0.01** |
| GDP per capita | Asia | Europe | -14652.86 | -37754.00 | 8448.29 | 1.00 |
|  |  | Latin America | 18159.80 | -13899.45 | 50219.04 | 0.98 |
|  |  | Africa | 26829.22 | -9899.26 | 63557.70 | 0.20 |
|  |  | NA and Oceania | -23557.51 | -60285.98 | 13170.97 | 0.78 |
|  | Europe | Latin America | 32812.65 | 4694.83 | 60930.47 | **0.01** |
|  |  | Africa | 41482.08 | 8138.45 | 74825.70 | **0.00** |
|  |  | NA and Oceania | -8904.65 | -42248.28 | 24438.98 | 1.00 |
|  | Latin America | Africa | 8669.43 | -31404.63 | 48743.48 | 1.00 |
|  |  | NA and Oceania | -41717.30 | -81791.35 | -1643.25 | **0.03** |
|  | Africa | NA and Oceania | -50386.73 | -94285.65 | -6487.80 | **0.01** |
| Education | Asia | Europe | -4.42 | -16.73 | 7.89 | 1.00 |
|  |  | Latin America | 10.58 | -6.51 | 27.67 | 1.00 |
|  |  | Africa | 20.13 | 0.55 | 39.71 | 0.18 |
|  |  | NA and Oceania | -19.82 | -39.40 | -0.24 | 0.14 |
|  | Europe | Latin America | 15.00 | 0.01 | 29.99 | 0.18 |
|  |  | Africa | 24.55 | 6.78 | 42.32 | **0.01** |
|  |  | NA and Oceania | -15.40 | -33.17 | 2.37 | 0.35 |
|  | Latin America | Africa | 9.55 | -11.81 | 30.91 | 1.00 |
|  |  | NA and Oceania | -30.40 | -51.76 | -9.04 | **0.01** |
|  | Africa | NA and Oceania | -39.95 | -63.35 | -16.55 | **0.00** |
| Trust | Asia | Europe | -11.51 | -27.91 | 4.89 | 1.00 |
|  |  | Latin America | 17.05 | -7.09 | 41.19 | 0.36 |
|  |  | Africa | 11.97 | -17.04 | 40.98 | 1.00 |
|  |  | NA and Oceania | -18.03 | -44.11 | 8.05 | 0.51 |
|  | Europe | Latin America | 28.56 | 7.03 | 50.08 | **0.00** |
|  |  | Africa | 23.48 | -3.40 | 50.35 | 0.17 |
|  |  | NA and Oceania | -6.52 | -30.20 | 17.15 | 1.00 |
|  | Latin America | Africa | -5.08 | -37.27 | 27.11 | 1.00 |
|  |  | NA and Oceania | -35.08 | -64.65 | -5.51 | **0.01** |
|  | Africa | NA and Oceania | -30.00 | -63.66 | 3.66 | 0.08 |
| Hospital beds | Asia | Europe | 0.95 | -1.66 | 3.56 | 1.00 |
|  |  | Latin America | 3.13 | -0.49 | 6.76 | 0.33 |
|  |  | Africa | 3.55 | -0.60 | 7.70 | 0.19 |
|  |  | NA and Oceania | 2.45 | -1.70 | 6.60 | 1.00 |
|  | Europe | Latin America | 2.19 | -0.99 | 5.36 | 0.05 |
|  |  | Africa | 2.60 | -1.17 | 6.37 | **0.04** |
|  |  | NA and Oceania | 1.50 | -2.27 | 5.27 | 1.00 |
|  | Latin America | Africa | 0.42 | -4.12 | 4.95 | 1.00 |
|  |  | NA and Oceania | -0.69 | -5.22 | 3.85 | 1.00 |
|  | Africa | NA and Oceania | -1.10 | -6.06 | 3.86 | 1.00 |

* P-values achieved using the Kruskal Wallis test

Supplementary table 4: Public health measures implemented by each country: time in days from 100 cases and time in days from 11/3/2020 (date of "pandemic" declaration by the WHO)

| **Country** | **Closure of primary schools** | | **Closure of secondary schools** | | **Selective entry restrictions** | | **Complete border closure** | | **Cancellation of mass events** | | **Social gatherings limited to less than 100** | | **Social gatherings limited to less than 10 people** | | **Restaurants and entertainment venues closure** | | **Non-essential business closure** | | **Lockdown** | |
| --- | --- | --- | --- | --- | --- | --- | --- | --- | --- | --- | --- | --- | --- | --- | --- | --- | --- | --- | --- | --- |
| *(Days from)* | 100 cases | Pan- demic | 100 cases | Pan- demic | 100 cases | Pan- demic | 100 cases | Pan- demic | 100 cases | Pan- demic | 100 cases | Pan- demic | 100 cases | Pan- demic | 100 cases | Pan- demic | 100 cases | Pan- demic | 100 cases | Pan- demic |
| **Argentina** | -5 | 4 | -5 | 4 | n/i | n/i | -4 | 5 | -8 | 1 | -1 | 8 | -1 | 8 | -1 | 8 | -1 | 8 | -1 | 8 |
| **Australia** | n/i | n/i | n/i | n/i | -38 | -39 | 9 | 8 | 8 | 7 | 8 | 7 | 19 | 18 | 12 | 11 | n/i | n/i | 14 | 13 |
| **Austria** | 7 | 5 | 7 | 5 | 0 | -2 | 8 | 6 | 1 | -1 | 7 | 5 | 7 | 5 | 7 | 5 | 7 | 5 | 7 | 5 |
| **Belgium** | 9 | 2 | 9 | 2 | n/i | n/i | 13 | 6 | 9 | 2 | 13 | 6 | 13 | 6 | 9 | 2 | 13 | 6 | 13 | 6 |
| **Brazil** | -2 | 2 | -2 | 2 | n/i | n/i | 12 | 16 | -2 | 2 | n/i | n/i | n/i | n/i | 6 | 10 | 6 | 10 | n/i | n/i |
| **Canada** | 0 | 1 | 0 | 1 | n/i | n/i | 6 | 7 | 5 | 6 | 5 | 6 | 16 | 17 | 5 | 6 | 11 | 12 | n/i | n/i |
| **Chile** | -2 | 4 | -2 | 4 | n/i | n/i | -1 | 5 | -4 | 2 | -1 | 5 | n/i | n/i | 4 | 10 | n/i | n/i | n/i | n/i |
| **Colombia** | -3 | 5 | -3 | 5 | -6 | 2 | 3 | 11 | -7 | 1 | -1 | 7 | 3 | 11 | -1 | 7 | 3 | 11 | 3 | 11 |
| **Costa Rica** | -5 | 5 | -5 | 5 | n/i | n/i | -5 | 5 | -12 | -2 | -6 | 4 | n/i | n/i | -6 | 4 | 11 | 21 | n/i | n/i |
| **Czech Republic** | -2 | 0 | -2 | 0 | -39 | -37 | 1 | 3 | -3 | -1 | -3 | -1 | 10 | 12 | 1 | 3 | 1 | 3 | 3 | 5 |
| **Denmark** | 3 | 2 | 3 | 2 | n/i | n/i | 4 | 3 | -4 | -5 | 8 | 7 | n/i | n/i | 8 | 7 | 8 | 7 | n/i | n/i |
| **Egypt** | -2 | 3 | -2 | 3 | n/i | n/i | 0 | 5 | 5 | 10 | n/i | n/i | n/i | n/i | 8 | 13 | n/i | n/i | n/i | n/i |
| **Estonia** | -3 | 1 | -3 | 1 | n/i | n/i | 0 | 4 | -3 | 1 | -3 | 1 | 9 | 13 | n/i | n/i | n/i | n/i | n/i | n/i |
| **Finland** | 3 | 5 | 5 | 7 | n/i | n/i | 5 | 7 | -1 | 1 | 3 | 5 | n/i | n/i | 22 | 24 | n/i | n/i | n/i | n/i |
| **France** | 12 | 2 | 12 | 2 | n/i | n/i | 15 | 5 | 3 | -7 | 13 | 3 | 15 | 5 | 13 | 3 | 13 | 3 | 15 | 5 |
| **Germany** | 12 | 2 | 12 | 2 | n/i | n/i | 14 | 4 | 9 | -1 | 21 | 11 | 21 | 11 | 15 | 5 | n/i | n/i | n/i | n/i |
| **Greece** | -3 | -1 | -3 | -1 | n/i | n/i | 5 | 7 | -5 | -3 | 5 | 7 | 9 | 11 | 0 | 2 | 0 | 2 | 9 | 11 |
| **Hong Kong** | -38 | -46 | -38 | -46 | -37 | -45 | 21 | 13 | -38 | -46 | 25 | 17 | 25 | 17 | n/i | n/i | n/i | n/i | n/i | n/i |
| **Hungary** | -8 | 2 | -8 | 2 | -13 | -3 | -5 | 5 | -10 | 0 | 6 | 16 | 6 | 16 | -5 | 5 | 6 | 16 | 6 | 16 |
| **Iceland** | n/i | n/i | 0 | 2 | n/i | n/i | 6 | 8 | 0 | 2 | 0 | 2 | n/i | n/i | 9 | 11 | 9 | 11 | n/i | n/i |
| **India** | 7 | 13 | 7 | 13 | -41 | -35 | 1 | 7 | 7 | 13 | 7 | 13 | 7 | 13 | 7 | 13 | 7 | 13 | 7 | 13 |
| **Ireland** | -3 | 1 | -3 | 1 | n/i | n/i | n/i | n/i | -3 | 1 | 9 | 13 | 12 | 16 | 9 | 13 | 9 | 13 | 12 | 16 |
| **Israel** | **-2** | **1** | **-2** | **1** | **-42** | **-39** | **-5** | **-2** | **-10** | **-7** | **-3** | **0** | **11** | **14** | **1** | **4** | **5** | **8** | **5** | **8** |
| **Italy** | 9 | -7 | 9 | -7 | -25 | -41 | 14 | -2 | 9 | -7 | 14 | -2 | 14 | -2 | 13 | -3 | 14 | -2 | 14 | -2 |
| **Japan** | 6 | -12 | 6 | -12 | -21 | -39 | n/i | n/i | n/i | n/i | n/i | n/i | n/i | n/i | n/i | n/i | n/i | n/i | n/i | n/i |
| **Kenya** | -19 | 4 | -19 | 4 | n/i | n/i | -19 | 4 | -21 | 2 | -21 | 2 | -21 | 2 | n/i | n/i | n/i | n/i | n/i | n/i |
| **Korea, South** | 10 | -9 | 10 | -9 | -19 | -38 | n/i | n/i | 0 | -19 | n/i | n/i | n/i | n/i | 29 | 10 | n/i | n/i | n/i | n/i |
| **Latvia** | -8 | 2 | -8 | 2 | n/i | n/i | -7 | 3 | -8 | 2 | -8 | 2 | 8 | 18 | n/i | n/i | n/i | n/i | n/i | n/i |
| **Lithuania** | -8 | 3 | -8 | 3 | n/i | n/i | -8 | 3 | -10 | 1 | -8 | 3 | -8 | 3 | -8 | 3 | -8 | 3 | -8 | 3 |
| **Luxembourg** | -6 | 1 | -6 | 1 | n/i | n/i | n/i | n/i | -6 | 1 | -3 | 4 | -3 | 4 | -3 | 4 | -3 | 4 | n/i | n/i |
| **Mexico** | -5 | 3 | -5 | 3 | 1 | 9 | n/i | n/i | -2 | 6 | 11 | 19 | n/i | n/i | 11 | 19 | 11 | 19 | n/i | n/i |
| **Netherlands** | 8 | 4 | 8 | 4 | 6 | 2 | 10 | 6 | 5 | 1 | 16 | 12 | 16 | 12 | 8 | 4 | n/i | n/i | n/i | n/i |
| **New Zealand** | 0 | 12 | 0 | 12 | -50 | -38 | -4 | 8 | -7 | 5 | 0 | 12 | 0 | 12 | 0 | 12 | 0 | 12 | 2 | 14 |
| **Norway** | 5 | 1 | 5 | 1 | n/i | n/i | 8 | 4 | 4 | 0 | 5 | 1 | 5 | 1 | 5 | 1 | n/i | n/i | n/i | n/i |
| **Poland** | -4 | 0 | -4 | 0 | n/i | n/i | -2 | 2 | -5 | -1 | -2 | 2 | 9 | 13 | -2 | 2 | 17 | 21 | 9 | 13 |
| **Portugal** | -2 | 1 | -2 | 1 | -4 | -1 | 3 | 6 | 1 | 4 | 5 | 8 | 5 | 8 | 5 | 8 | 5 | 8 | 5 | 8 |
| **Russia** | 1 | 8 | 1 | 8 | -48 | -41 | -2 | 5 | 1 | 8 | 7 | 14 | 7 | 14 | 7 | 14 | 7 | 14 | 7 | 14 |
| **Rwanda** | -22 | 3 | -22 | 3 | n/i | n/i | -16 | 9 | -22 | 3 | -15 | 10 | -15 | 10 | -15 | 10 | -15 | 10 | -15 | 10 |
| **Singapore** | 33 | 23 | 33 | 23 | -38 | -48 | 21 | 11 | 12 | 2 | 23 | 13 | 37 | 27 | 33 | 23 | 33 | 23 | n/i | n/i |
| **Slovakia** | -6 | 2 | -6 | 2 | n/i | n/i | -6 | 2 | -10 | -2 | -7 | 1 | 18 | 26 | -4 | 4 | -4 | 4 | 18 | 26 |
| **Slovenia** | -2 | 1 | -2 | 1 | -4 | -1 | 1 | 4 | -7 | -4 | 5 | 8 | 5 | 8 | 1 | 4 | 1 | 4 | 15 | 18 |
| **South Africa** | -4 | 4 | -4 | 4 | -4 | 4 | -1 | 7 | -4 | 4 | -1 | 7 | -1 | 7 | -1 | 7 | -1 | 7 | -1 | 7 |
| **Spain** | 10 | 1 | 10 | 1 | 8 | -1 | 20 | 11 | 12 | 3 | 12 | 3 | 12 | 3 | 12 | 3 | 12 | 3 | 12 | 3 |
| **Sweden** | n/i | n/i | 10 | 6 | -5 | -9 | 11 | 7 | 4 | 0 | 20 | 16 | n/i | n/i | n/i | n/i | n/i | n/i | n/i | n/i |
| **Switzerland** | 6 | 2 | 6 | 2 | 6 | 2 | 10 | 6 | -8 | -12 | 6 | 2 | 13 | 9 | 9 | 5 | 9 | 5 | n/i | n/i |
| **Taiwan** | -46 | -38 | -46 | -38 | -53 | -45 | -1 | 7 | 6 | 14 | n/i | n/i | n/i | n/i | n/i | n/i | n/i | n/i | n/i | n/i |
| **Thailand** | 1 | 6 | 1 | 6 | n/i | n/i | 16 | 21 | 10 | 15 | 10 | 15 | n/i | n/i | 10 | 15 | n/i | n/i | n/i | n/i |
| **Turkey** | -7 | 1 | -7 | 1 | -56 | -48 | 8 | 16 | 7 | 15 | n/i | n/i | n/i | n/i | 2 | 10 | 7 | 15 | n/i | n/i |
| **United Kingdom** | 14 | 7 | 14 | 7 | n/i | n/i | n/i | n/i | 19 | 12 | 19 | 12 | 19 | 12 | 15 | 8 | 19 | 12 | 19 | 12 |
| **United States** | 16 | 8 | 16 | 8 | -32 | -40 | n/i | n/i | 8 | 0 | 16 | 8 | 16 | 8 | 12 | 4 | 16 | 8 | 16 | 8 |

n/i= not implemented

Supplementary table 5: Comparison of rates and percentage of countries implementing each measure in the OECD nations versus non-OECD nations

| **Measure/Pre-pandemic characteristic** | **All countries (N=50)**  **N (%)** | **OECD (N=38)**  **n (%)** | **Non-OECD (N=12)**  **n (%)** | **P-value** |
| --- | --- | --- | --- | --- |
| Primary school closure | 47 (94%) | 35 (92.1%) | 12 (100%) | 0.560 |
| Secondary schools | 49 (98%) | 37 (97.4%) | 12 (100%) | 1.000 |
| Selective entry restrictions | 25 (50%) | 19 (50%) | 6 (50%) | 1.000 |
| Border closure | 43 (86%) | 31 (81.6%) | 12 (100%) | 0.234 |
| Cancellation mass events | 49 (98%) | 37 (97.4% | 12 (100%) | 1.000 |
| Social gatherings limited to less than 100 | 44 (88%) | 35 (92.1%) | 9 (75%) | 0.141 |
| Social gatherings limited to less than 10 | 36 (72%) | 28 (73.7%) | 8 (66.7%) | 0.718 |
| Restaurants closed | 43 (86%) | 34 (89.5%) | 9 (75%) | 0.337 |
| Non-essential businesses closed | 34 (68%) | 27 (71.1%) | 7 (58.3%) | 0,486 |
| Lockdown | 25 (50%) | 20 (52.6%) | 5 (41.7%) | 0.508 |

Supplementary table 6: Categorical association between implementation of measures and high/low level of: GDP, trust, Democracy Index and number of hospital beds per population:

| **Measure/Pre-pandemic characteristic** | **High education level n=21** including Israel | **Low education level  n=29** | **P-value** | **High democracy level^a^ (n=19)** | **Low democracy level  (n=31)^a^ i**ncluding Israel | **P-value** | **High GDP^b^  (n=21)** including Israel | **Low GDP^b^  (n=29)** | **P-value** | **High trust index^c^ (n=20)** | **Low trust index^c^  (n=28)** including Israel | **P-value** | **High # of hospital beds per 1,000^d^  (n=23)** | **Low # of hospital beds per 1,000^d^  (n=27)** including Israel | **P-value** |
| --- | --- | --- | --- | --- | --- | --- | --- | --- | --- | --- | --- | --- | --- | --- | --- |
|  | n (%) | n (%) |  | n (%) | n (%) |  | n (%) | n (%) |  | n (%) | n (%) |  | n (%) | n (%) |  |
| Primary school closure | 19 (90.5%) | 28 (96.6%) | 0.57 | 16 (84.2%) | 31 (100%) | 0.09 | 18 (85.7% | 29 (100%) | 0.12 | 17 (85%) | 28 (100%) | 0.12 | 22 (95.7%) | 25 (92.6%) | 1.00 |
| Secondary schools | 20 (95.2%) | 29 (100%) | 0.38 | 18 (94.7%) | 31 (100%) | 0.33 | 20 (95.2%) | 29 (100%) | 0.38 | 19 (95%) | 28 (100%) | 0.37 | 22 (95.7%) | 27 (100%) | 0.43 |
| Selective entry restrictions | 10 (47.6%) | 15 (51.7%) | 0.78 | 7 (36.8%) | 18 (58.1%) | 0.15 | 10 (47.6%) | 15 (51.7%) | 0.77 | 10 (50%) | 15 (53.6%) | 0.81 | 11 (47.8%) | 14 (51.9%) | 0.78 |
| Border closure | 15 (71.4%) | 28 (96.6%) | 0.03 | 16 (84.2%) | 27 (87.1%) | 1.00 | 17 (81%) | 26 (89.7%) | 0.42 | 17 (85%) | 24 (85.7%) | 1.00 | 20 (87%) | 23 (85.2%) | 1.00 |
| Cancellation mass events | 20 (95.2%) | 29 (100%) | 0.38 | 19 (100%) | 30 (96.8%) | 0.70 | 21 (100%) | 28 (96.6%) | 0.62 | 20 (100%) | 27 (96.4%) | 0.63 | 22 (95.7%) | 27 (100%) | 0.43 |
| Social gatherings limited to less than 100 | 18 (85.7%) | 26 (89.7%) | 0.69 | 19 (100%) | 25 (80.7%) | 0.13 | 21 (100%) | 23 (79.3%) | 0.10 | 20 (100%) | 22 (78.6%) | 0.10 | 20 (87%) | 24 (88.9%) | 1.00 |
| Social gatherings limited to less than 10 | 15 (71.4%) | 21 (72.4%) | 0.94 | 13 (68.4%) | 23 (74.2%) | 0.66 | 17 (81%) | 19 (65.5%) | 0.23 | 16 (80%) | 19 (67.9%) | 0.35 | 19 (82.6%) | 17 (63%) | 0.12 |
| Restaurants closed | 16 (76.2%) | 27 (93.1%) | 0.06 | 18 (94.7%) | 25 (80.6%) | 0.70 | 19 (90.55) | 24 (82.8%) | 1.00 | 17 (85%) | 25 (89.3%) | 0.68 | 19 (82.6%) | 24 (88.9%) | 0.44 |
| Non-essential businesses closed | 11 (52.4%) | 23 (79.3%) | 0.04 | 12 (63.2%) | 22 (71%) | 0.57 | 14 (66.7%) | 20 (69%) | 0.86 | 12 (60%) | 21 (75%) | 0.27 | 14 (60.9%) | 20 (74.1%) | 0.32 |
| Lockdown | 7 (33.3%) | 18 (62.1%) | **0.045** | 7 (36.8%) | 18 (58.1%) | 0.15 | 9 (42.9%) | 16 (55.2%) | 0.39 | 8 (40%) | 17 (60.7%) | 0.16 | 14 (60.9%) | 11 (40.7%0 | 0.16 |

^a^High democracy level- >8.055; Low democracy level- </=8.055,

^b^High GDP- > 40,370.4 USD; Low GDP- </=40,370.4 USD

^c^High trust index- >33.70; Low trust index- </=33.70

^d^High # of hospital beds per 1,000- >3.31; Low # of hospital beds per 1,000- </=3.31

Supplementary table 7: Full Cox proportional hazards model of: nationwide lockdown (7a), primary school closure (7b), secondary school closure (7c); and restaurant and entertainment venues closure (7d); by pre-pandemic characteristics and geographic location

**Table 7a**: **Nationwide lockdown**

| **Variable** | **Category** | **Hazard Ratio** | **95% CI** | **p value** |
| --- | --- | --- | --- | --- |
| **Geographic location** | Other  Asia  Europe | 1  0.241  1.103 | -  .051-1.143  .453-2.686 | -  .073  .829 |
| **Democracy** | Low (ref)  High | 1  0.348 | -  .139-.875 | -  **.025** |
| **Education** | Low (ref)  High | Not entered in model | - | .162 |
| **GDP** | Low (ref)  High | Not entered in model | - | .952 |
| **Trust** | Low (ref)  High | Not entered in model | - | .437 |

**Table 7b**: **Primary school** **closure**

| **Variable** | **Category** | **Hazard Ratio** | **95% CI** | **p value** |
| --- | --- | --- | --- | --- |
| **Geographic location** | Other  Asia  Europe | 1  .443  .734 | -  .171-1.148  .365-1.479 | -  .094  .387 |
| **GDP** | Low (ref)  High | 1  .246 | -  .119-.507 | -  **<****0.001** |
| **Education** | Low (ref)  High | Not entered in model | - | .576 |
| **Trust** | Low (ref)  High | Not entered in model | - | .071 |
| **Democracy** | Low (ref)  High | Not entered in model | - | .110 |

**Table 7c**: S**econdary school** **closure**

| **Variable** | **Category** | **Hazard Ratio** | **95% CI** | **p value** |
| --- | --- | --- | --- | --- |
| **Geographic location** | Other  Asia  Europe | 1  .466  .929 | -  .181-1.201  .457-1.890 | -  .114  .840 |
| **GDP** | Low (ref)  High | 1  .279 | -  .141-.554 | -  **<0.001** |
| **Education** | Low (ref)  High | Not entered in model | - | .991 |
| **Trust** | Low (ref)  High | Not entered in model | - | .073 |
| **Democracy** | Low (ref)  High | Not entered in model | - | .176 |

**Table 7d**: R**estaurants and entertainment venues** **closure**

| **Variable** | **Category** | **Hazard Ratio** | **95% CI** | **p value** |
| --- | --- | --- | --- | --- |
| **Geographic location** | Other  Asia  Europe | 1  .174  .481 | -  .06-.53  .24-.97 | -  0.02  0.042 |
| **Education** | Low (ref)  High | 1  .361 | -  .18-.72 | -  .**004** |
| **Democracy** | Low (ref)  High | Not entered in model | - | .526 |
